# Laparoscopic or open liver resection for intrahepatic cholangiocarcinoma: A meta-analysis and systematic review

**DOI:** 10.3389/fonc.2023.1096714

**Published:** 2023-03-01

**Authors:** Xin Zhao, Feng-wei Gao, Kang-yi Jiang, Jie Yang, Qing-yun Xie, Jie Gong, Man-yu Yang, Tian-yang Mao, Ze-hua Lei

**Affiliations:** ^1^ Department of Hepatopancreatobiliary Surgery, The People’s Hospital of Leshan, Leshan, Sichuan, China; ^2^ Diagnosis and Treatment Center for Liver, Gallbladder, Pancreas and Spleen System Diseases of Leshan, Leshan, Sichuan, China; ^3^ North Sichuan Medical College, Nanchong, Sichuan, China

**Keywords:** intrahepatic cholangiocarcinoma, laparotomy, laparoscopy, hepatectomy, meta-analysis

## Abstract

**Background:**

Although laparoscopic hepatectomy has been widely used in the treatment of benign and malignant liver diseases, its applicability in intrahepatic cholangiocarcinoma (ICC) is controversial. We conducted a meta-analysis to compare the short-term and long-term outcomes of laparoscopic hepatectomy (Lap-ICC) and open hepatectomy (Open-ICC) in ICC patients.

**Methods:**

The PubMed, Web of science, Cochrane Library, China National Knowledge Infrastructure and other databases were searched for the relevant literature. The research data were extracted according to the inclusion and exclusion criteria.

**Results:**

Seventeen studies, including 3975 ICC patients, were selected for the meta-analysis. Compared to Open-ICC, Lap-ICC had lower rates of lymph node dissection (OR=0.44, P=0.01) and metastasis (OR=0.58, P=0.03), along with less intraoperative bleeding (MD=-128.43 ml, P<0.01) lower blood transfusion rate (OR=0.43, P<0.01), shorter hospital stay (MD=-2.75 day, P<0.01), higher R0 resection rate (OR=1.60, P<0.01), and lower tumor recurrence rate (OR=0.67, P=0.01). However, there was no difference between the two groups in terms of operation time, number of lymph node dissection, incision margin distance, overall complications rate, severe complications rate, and the 1-, 3- and 5-year DFS and OS rates.

**Conclusion:**

Laparoscopic hepatectomy is partially superior to open hepatectomy in terms of less bleeding, shorter hospital stay and higher R0 resection rate, while the long-term efficacy of the two approaches is similar.

## Introduction

Intrahepatic cholangiocarcinoma (ICC) is the second most common malignancy of the liver, and its incidence is increasing on a yearly basis worldwide. According to the data reported in the United States, the incidence of ICC increased by about 5.9% annually from 2003 to 2009 (1–3). Recent advances in our knowledge of the mechanism of ICC have not translated to improved treatment strategies (4–6). Surgical resection and chemotherapy are the most common therapeutic modalities for ICC, and there have been some reports on immunotherapy as well (3, 7).

Hepatectomy, including the open, laparoscopic and robotic forms, is the most accurate curative option for ICC, especially for single lesions. Studies show that while the short-term outcomes of laparoscopic hepatectomy (Lap-ICC) are superior to that of open hepatectomy (Open-ICC), the long-term effects are similar (8–11). Unlike hepatocellular carcinoma (HCC), ICC often requires lymph node dissection. However, laparoscopic lymph node dissection is difficult, and beset with problems such as insufficient dissection and inaccurate tumor staging (12, 13). Therefore, it remains to be ascertained whether Lap-ICC is indeed better than Open-ICC.

Guerrini et al. conducted a meta-analysis of four studies, and found that the short-term efficacy of Lap-ICC was better than that of Open-ICC. In contrast, Wei et al. reported similar short-term and long-term outcomes of both approaches in a meta-analysis of six studies (14, 15). The meta-analyses conducted by Machairas et al., Ziogas et al. and Regmi on eight identical studies also differed in their results (16–18), which can be attributed to the limited number and quality of the included literature, as well as the lack of further subgroup analysis. To this end, we conducted a meta-analysis to compare the short-term and long-term outcomes of Lap-ICC and Open-ICC in a large cohort of ICC patients, and performed subgroup analysis to further assess the reliability of the results.

## Methods

### Search strategy

This study was conducted in accordance with the systematic review and meta-analysis (PRISMA) (19) and the guidelines for evaluating the methodological quality of systematic review (AMSTAR), and has been registered in PROPERO with the registration number CRD23457688. The PubMed, Web of science, EMbase, The Cochrane Library and CNKI databases were searched for randomized controlled trials (RCTs) and non-RCTs published till June 30, 2022 that compared the short-term and long-term efficacy of Lap-ICC and Open-ICC. The search keywords were “intrahepatic cholangiocarcinoma”, “laparoscopy”, “liver reservation”, “hepatectomy”, etc. The reference lists of the selected articles were manually searched for additional studies.

### Inclusion and exclusion criteria

The inclusion criteria for the studies were as follows: (1) RCTs and well-designed non-RCTs, (2) comparison of Lap-ICC and Open-ICC groups, (3) outcome indicators such as operation time, intraoperative blood loss, blood transfusion rate, R0 resection rate, overall complication rate, postoperative hospital stay, tumor recurrence rate, and 1-, 3- and 5-year disease-free survival (DFS) and overall survival (OS) rates, (4) literature quality evaluated as medium low risk bias and NOS score ≥ 5, and (5) published in Chinese or English.

The exclusion criteria were as follows: (1) summaries, case reports, minutes of meeting and other articles, (2) lack of control group or absence of outcome indicators, and (3) highly biased according to The Cochrane Bias Risk Assessment Form and non-RCT with Ottawa Scale (NOS) scores below 5.

### Data extraction

Two researchers extracted the data according to the preset form, and rechecked the data in case of any inconsistency. Any disputes were settled by discussing with a third researcher. The following data were extracted: (1) general information, including title, author, publication date, country, etc., (2) general data of subjects such as number of cases, male/female ratio, age, BMI, tumor diameter, etc., and (3) outcome indicators such as operation time, intraoperative blood loss, intraoperative lymph node dissection rate, blood transfusion rate, overall complication rate, serious complication rate, bile leakage rate, hospital stay, R0 resection rate, tumor recurrence rate, and the 1-, 3- and 5-year OS and DFS rates.

### Quality assessment

The quality of the non-RCTs was evaluated by two researchers using the NOS. The risk of bias in the RCTs was assessed as per the recommendations in Version 5.1.0 of the Cochrane System Evaluator’s Manual. In case of differences, a third researcher re-evaluated the studies.

### Statistical analysis

The Revman 5.3 software of the Cochrane Center was used for statistical analysis. Odd ratio (OR) and 95% confidence interval (CI) were used as indicators for the counting data, and the mean difference (MD) and 95% confidence interval (CI) were used for measurement data. When only median and extreme values were reported in the study, the method proposed by Hozo et al. was used (20) to estimate the mean and standard deviation (SD). The heterogeneity among the studies was analyzed by Q test and I^2^ test. The fixed effect model was used to analyze studies with low heterogeneity (I^2^ < 50%), whereas the random effect model was used in case of high heterogeneity (I^2^≥ 50%). Subgroup analysis was performed as required. When the results of non-subgroup analyses were statistically consistent with the results of subgroup analyses, the former was considered. The PSM subgroups were used in case of any differences. The publication bias was evaluated by a funnel chart. All tests were two-sided and P < 0.05 was considered statistically significant.

## Results

### Study selection

**Figure 1:**
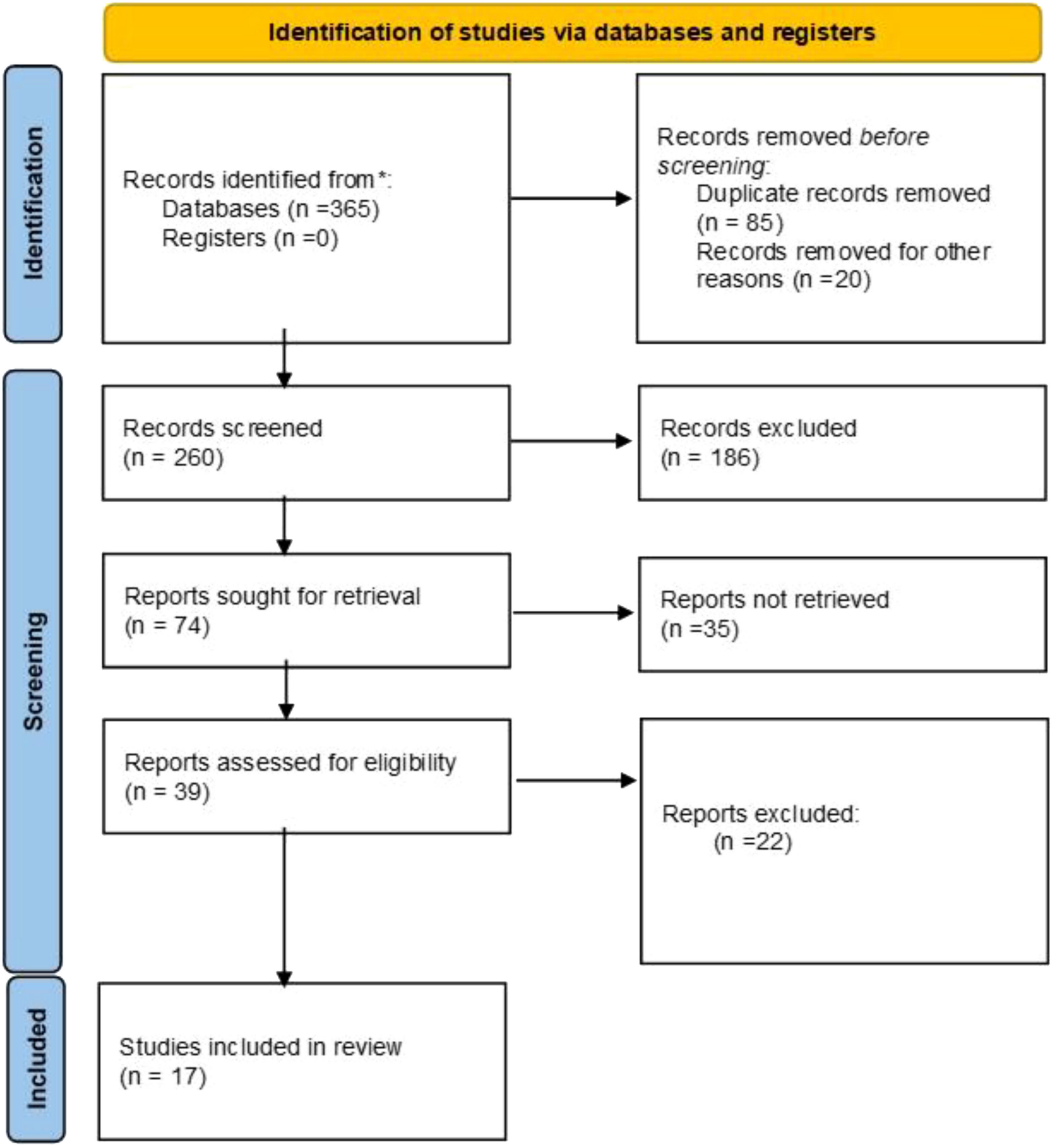
The PRISMA diagram for the selection of the studies.

A total of 365 articles were retrieved from the initial screening. Seventeen articles (21–37), including 15 that were published in English and 2 in Chinese, were incorporated into the meta-analysis. There were 8 propensity matching score (PSM) studies and 9 retrospective studies which unused propensity matching score, including a total of 3975 patients (1083 in the Lap-ICC group and 2892 in the Open-ICC group). The flow chart of study selection and the results of the meta-analysis are summarized in **Figure 1**. The basic characteristics and NOS scores of the included studies are summarized in **Table 1**.

**Table 1:** The basic characteristics and NOS scores.

First author	Year	Country	Study type	Study period	Operation	Cases	Age (years)	Sex (M/F)	BMI (kg/m2)	Tumor size (mm)	NOS
Uy et al (21)	2015	Korea	RS	2004-2012	Lap	11	67 (49–82)	9/2	N	41.5 (20–136)	5^a^
					Open	26	67 (45–84)	18/8	N	4.25 (12–140)	
Lee et al (22)	2016	Korea	RS	2010-2015	Lap	14	66 (47–78)	11/3	N	35 (7–5)	6^a^
					Open	23	59 (47–76)	19/4	N	4 (12–8)	
Wei et al (23)	2017	China	RS	2004-2016	Lap	12	N	N	N	5.25 (3–9)	5^a^
					Open	20	N	N	N	6 (0.7–13)	
Martin et al (24)	2019	America	RS	2010-2015	Lap	312	64.7	131/181	N	N	5^a^
					Open	1997	63.9	933/1064	N	N	
Zhu et al (25)	2019	China	PSM	2012-2017	Lap	18	54.1 ± 16.6	10/8	23.0 ± 3.4	60 (30–90)	6^a^
					Open	36	55.6 ± 9.8	19/17	23.4 ± 5.2	60 (30–90)	
Kinoshita et al (28)	2019	Japan	RS	2010-2018	Lap	15	65 ± 13	7/8	N	26 ± 16	6^a^
					Open	21	68 ± 8.6	16/5	N	34 ± 15	
Haber et al (26)	2020	Germany	RS	2015-2019	Lap	27	69 (44–83)	13/14	24.6 (17.7–31)	60 (14–132)	6^a^
					Open	31	63 (33–82)	18/13	24.6 (19–36.2)	65 (13–153)	
Kang et al (27)	2020	Korea	PSM	2004-2015	Lap	24	66.8 ± 9.7	15/9	N	47 ± 33	7^a^
					Open	24	68.1 ± 10.2	15/9	N	41 ± 18	
Wu et al (30)	2020	China	RS	2010-2017	Lap	18	64 (60-72)	12/6	N	N	6^a^
					Open	25	61 (55-64)	10/15	N	N	
Ratti et al (29)	2020	Italy	PSM	2004-2017	Lap	104	59 ± 5	70/34	24.1 ± 1.9	39 ± 17	7^a^
					Open	104	61 ± 6	68/36	24.6 ± 1.6	41 ± 12	
Lee et al (33)	2021	Korea	PSM	2004-2017	Lap	30	57.43 ± 10.03	22/8	N	39.3 ± 26.7	7^a^
					Open	30	56.27 ± 9.86	26/4	N	33.3 ± 14.6	
Hobeika et al (31)	2021	France	PSM	2000-2017	Lap	109	67 (60–72)	N	25.8 (23.0–29.3)	> 5 45	7^a^
					Open	109	61 (52–68)	N	25.8 (22.9–29.3)	> 5 45	
Chen et al (34)	2021	China	RS	2014-2020	Lap	23	56 ± 10	13/10	N	N	5^a^
					Open	42	49 ± 12	24/18	N	N	
Ma et al (35)	2021	China	RS	2015-2020	Lap	40	61.5 ± 9.1	18/22	23.4 ± 3.2	44 ± 18	6^a^
					Open	78	61.2 ± 8.3	48/30	23.2 ± 3.4	60 ± 33	
Brustia et al (36)	2022	France	PSM	2000-2018	Lap	89	65.24 ± 11.4	52/37	25.82 ± 4.55	46.7 ± 25.6	7^a^
					Open	89	67.92 ± 8.97	38/51	25.28 ± 4.40	53.2 ± 37.3	
Yang et al (7)	2022	China	PSM	2011-2018	Lap	122	>65y 43	54/68	23.6 (21.7-25.8)	43.5 (30-60)	6^a^
					Open	122	>65y 33	49/73	24.2 (21.4-27.2)	50.0 (35-60)	
Salehi et al (37)	2022	USA	PSM	2010-2016	Lap	115	65.7 (34–87)	42/73	N	42 (30–60)	7^a^
					Open	115	67.1 (40–85)	37/78	N	44 (30–70)	

a NOS; NA, not available; RS, Retrospective study; PSM, propensity score-matched analyses; BMI, Body mass index;

NOS, Newcastle-Ottawa scale.

### Meta-analysis results

The results of the meta-analysis are summarized in **Table 2**

**Table 2:** Summary of analysis results.

Outcomes	Study	Cases	I^2^	MD/OR (95% CI)	P value
Operating time	14	1258	83%	10.30 (-6.30, 26.91)	0.22
Blood loss	12	956	80%	-128.43 (-116.74, -89.83)	**<0.01**
Transfusion	9	1154	0	0.43 (0.31,0.58)	**<0.01**
Lymph node dissection	10	934	66%	0.44 (0.23,0.82)	**0.01**
Overall complications	11	906	45%	0.55 (0.41,0.75)	**<0.01**
Severe complications	11	1238	17%	0.77 (0.55,1.08)	0.13
Biliary leakage	6	518	0%	0.71 (0.33,1.53)	0.38
Hospital stay	12	1185	98%	-2.75 (-4.38, -1.12)	**<0.01**
R0	11	3618	0%	1.60 (1.28,1.99)	**<0.01**
Number of lymph node	6	764	98%	-1.46 (-2.94, 0.02)	0.05
Resection margin	5	515	71%	1.53 (-1.62,4.67)	0.34
Lymph node metastasis	7	460	11%	0.58 (0.36,0.94)	**0.03**
Recurrence	9	390	0	0.67 (0.49,0.92)	**0.01**
Intrahepatic recurrence	5	361	20%	0.68 (0.43,1.07)	0.09
Extrahepatic recurrence	5	361	1%	0.69 (0.40,1.17)	0.17
1 OS	10	1215	0%	1.00 (0.71,1.40)	0.99
3 OS	13	1251	0%	1.30 (1.00,1.68)	0.05
5 OS	6	769	0%	1.38 (0.92,2.07)	0.12
1 DFS	8	941	0%	1.20 (0.89,1.62)	0.22
3 DFS	11	977	20%	1.17 (0.87,1.56)	0.3
5 DFS	4	495	67%	0.79 (0.26,2.39)	0.68

MD/OR, mean difference/odd ratio.

Statistical significant results are shown in bold.

## Short-term outcomes

### Operating time

**Figure 2:**
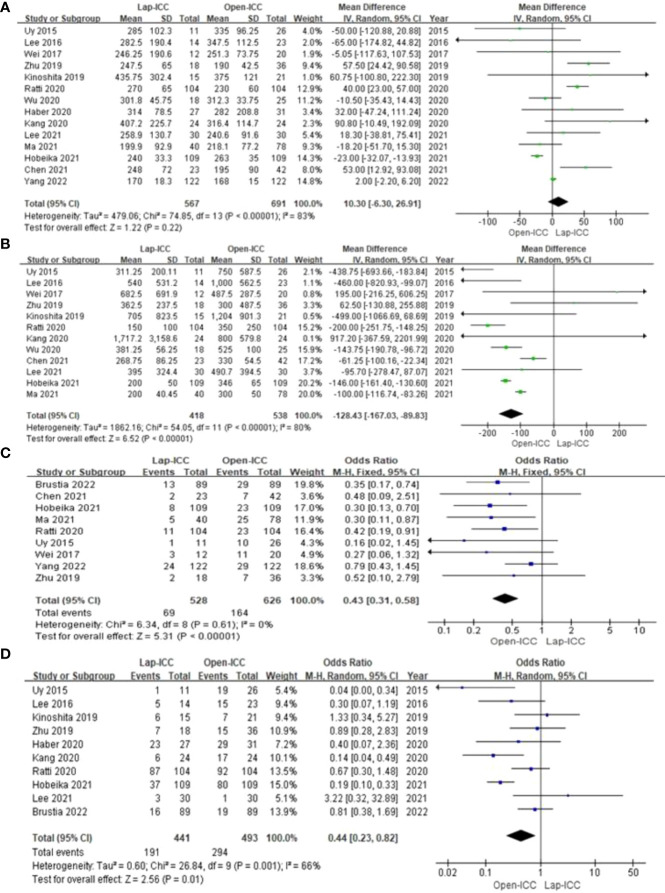
**(A)**operating time; **(B)** blood loss; **(C)** transfusion; **(D)** Lymph node dissection.

Fourteen studies reported the operation time. There was a high degree of heterogeneity among the studies (I^2^ = 83%), which warranted the random effect model. As shown in **Figure 2A**, there was no significant difference in the operation time between the two groups (MD=10.30, 95%CI= -6.30**~**26.91, P=0.22).

### Blood loss

Twelve studies reported the amount of intraoperative bleeding and showed significant heterogeneity (I^2^ = 80%). The random effect model analysis showed that intraoperative bleeding was significantly lower in the Lap-ICC group compared to that in the Open-ICC group (MD=-128.43, 95%CI= -116.74**~**-89.83, P<0.01) (**Figure 2B**).

### Transfusion

Nine studies reported blood transfusion rates. The heterogeneity was insignificant (I^2^ = 0%) and the fixed effect model was used. As shown in **Figure 2C**, the rate of perioperative blood transfusion was significantly lower in the Lap-ICC group compared to that in Open-ICC group (OR=0.43, 95%CI= 0.31**~**0.58, P<0.01).

### Lymph node dissection

Ten studies reported lymph node dissection and there was a high degree of heterogeneity (I^2^ = 66%). Using the random effect model, we found that the rate of lymph node dissection was significantly lower in the Lap-ICC group compared to that in the Open-ICC group (OR=0.44, 95%CI= 0.23**~**0.82, P=0.01) (**Figure 2D**).

### Overall complications

**Figure 3:**
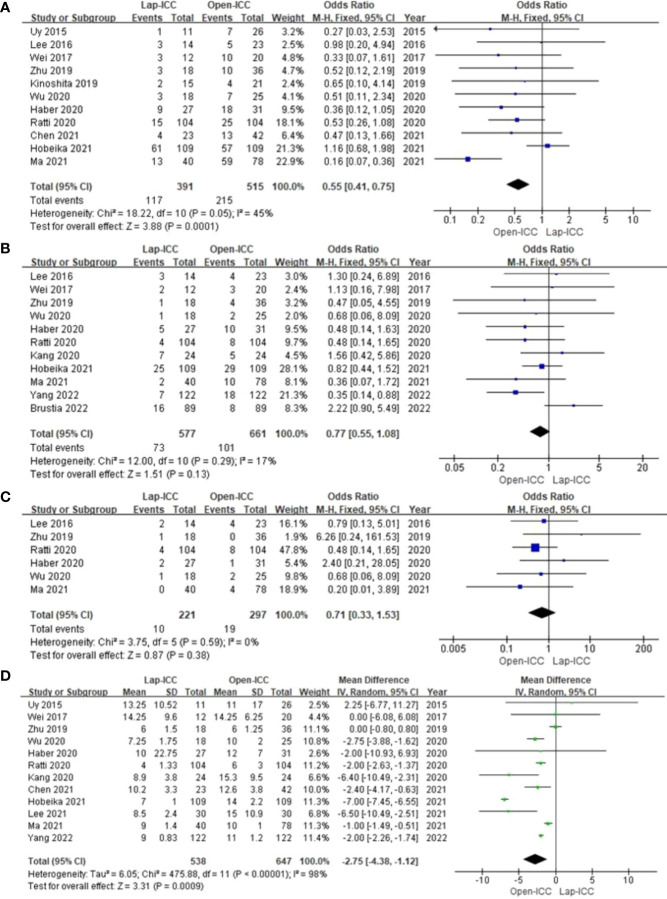
**(A)** overall complications; **(B)** severe complications; **(C)** biliary leakage; **(D)** hospital stay.

Eleven studies reported overall complications, and no obvious heterogeneity was observed (I^2^ = 45%). The fixed effect model analysis showed that the Lap-ICC group had significantly lower overall complications than that in the Open-ICC group (OR=0.55, 95%CI= 0.41**~**0.75, P<0.01) (**Figure 3A**).

### Severe complications (Clavien Dindo classification > II)

Eleven studies reported severe complications without any significant heterogeneity (I^2^ = 17%). Fixed effect model analysis did not show any significant difference between the two groups (OR=0.77, 95%CI= 0.55**~**1.08, P=0.13) (**Figure 3B**).

### Biliary leakage

Six studies reported biliary leakage. The heterogeneity among the studies was low (I^2^ = 0%) and the fixed effect model did not reveal any significant differences (OR=0.71, 95%CI= 0.33**~**1.53, P=0.38) (**Figure 3C**).

### Hospital stay

Twelve studies reported the duration of hospital stay, and there was significant heterogeneity among the results (I^2^ = 98%). The random effect model analysis showed that Lap-ICC was associated with a significantly shorter hospital stay compared to Open-ICC (MD=-2.75, 95%CI= -4.38**~**-1.12, P<0.01) (**Figure 3D**).

## Pathological features

### R0

**Figure 4:**
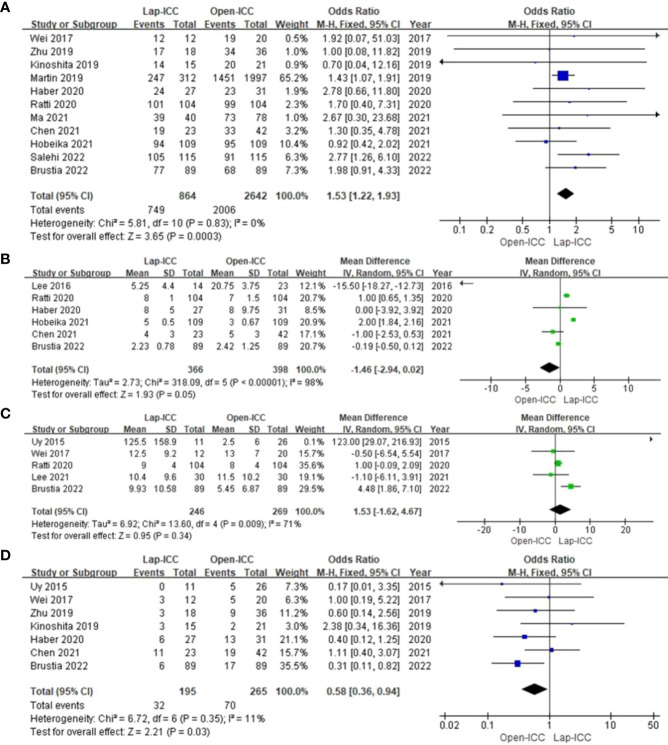
**(A)** R0; **(B)** number of lymph nodes; **(C)** resection margin; **(D)** Lymph node metastasis.

Eleven studies reported the R0 resection rate, and the heterogeneity was low (I^2^ = 0%). Fixed effect model analysis showed that the rate of R0 in the Lap-ICC group was significantly higher than that in the Open-ICC group (OR=1.60, 95%CI= 1.28**~**1.99, P<0.01) (**Figure 4A**).

### Number of lymph nodes

Six studies reported the number of lymph nodes (I^2^ = 98%). There was a high degree of heterogeneity among the studies, and the random effect model analysis showed that there was almost significant difference between the two groups (MD=-1.46, 95%CI= -2.94**~**0.02, P=0.05) (**Figure 4B**).

### Resection margin

Five studies reported the resection margin with a high degree of heterogeneity (I^2^ = 71%). The random effect model analysis showed that there was no statistically significant difference between the two groups (MD=-1.53, 95%CI= -1.62**~**4.67, P=0.34) (**Figure 4C**).

### Lymph node metastasis

Seven studies reported lymph node metastasis. The heterogeneity was low (I^2^ = 11%) and the fixed effect model showed that lymph node metastasis in the Lap-ICC group was significantly lower than that in the Open-ICC group (OR=0.58, 95%CI= -0.36**~**0.94, P=0.03) (**Figure 4D**).

## Long-term outcomes

### Recurrence

**Figure 5:**
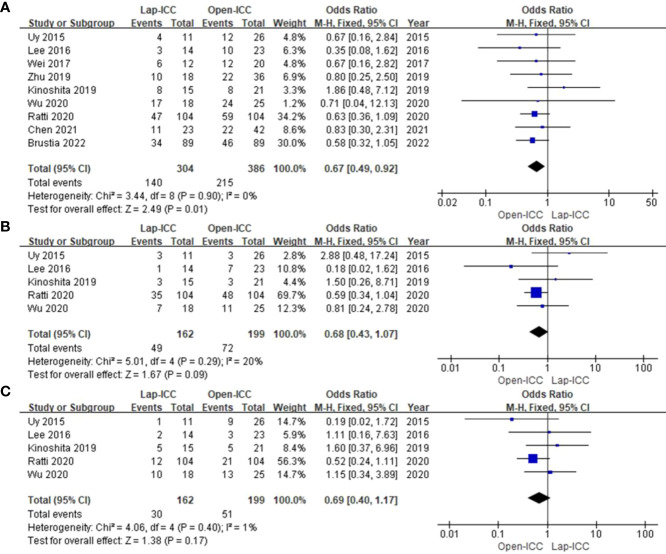
**(A)** recurrence; **(B)** intrahepatic recurrence; **(C)** extrahepatic recurrence.

Nine studies reported recurrence and the heterogeneity was low (I^2^ = 0%). Fixed effect model analysis revealed significantly lower recurrence rates in the Lap-ICC group compared to that in Open-ICC group (OR=0.67, 95%CI= 0.49~0.92, P=0.01) (**Figure 5A**). In addition, 5 studies reported intrahepatic (OR=0.68, 95%CI= 0.43~1.07, P=0.09) (**Figure 5B**) and extrahepatic recurrences (OR=0.69, 95%CI= 0.40~1.17, P=0.17), no significant differences were observed between the two groups (**Figure 5C**).

### DFS

**Figure 6:**
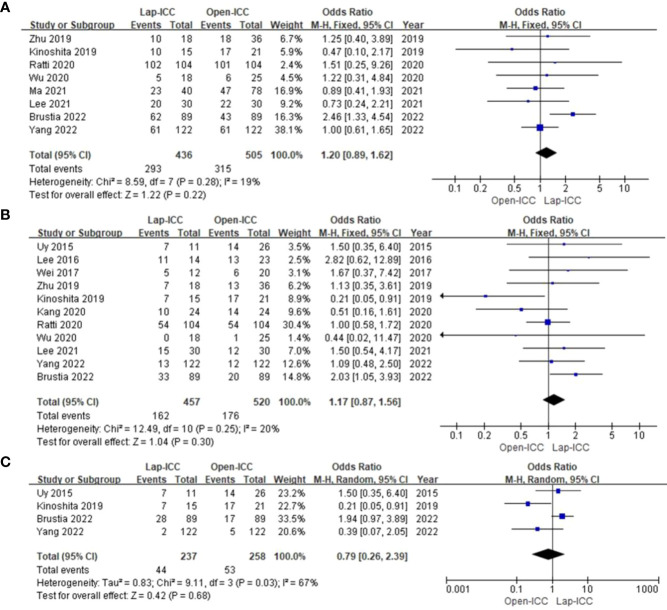
**(A)** 1-year DFS; **(B)** 3-year DFS; **(C)** 5-year DFS.

Eight studies reported 1-year DFS, 11 studies reported 3-year DFS and 4 studies reported 5-year DFS. The heterogeneity among studies reporting 1-year DFS and 3-year DFS was low (I^2^ = 0% and I^2^ = 20%), whereas high heterogeneity (I^2^ = 67%) was observed for studies reporting 5-year DFS. The respective models indicated that the 1-year DFS (OR=1.20, 95%CI= 0.89~1.62, P=0.22) (**Figure 6A**), 3-year DFS (OR=1.17, 95%CI= 0.87**~**1.56, P=0.3) (**Figure 6B**) and 5-year DFS (OR=0.79, 95%CI= 0.26**~**2.39, P=0.69) were similar in both groups (**Figure 6C**).

### OS

**Figure 7:**
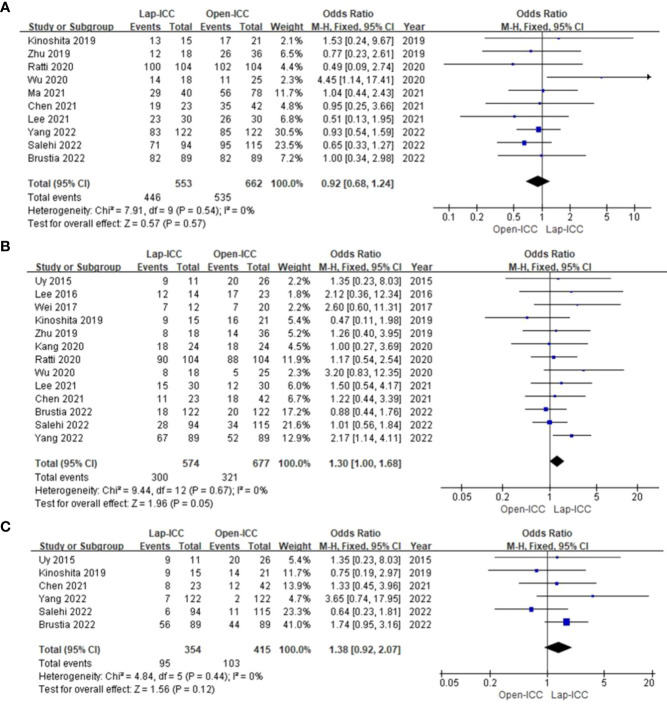
**(A) **1-year OS; **(B)** 3-year OS; **(C)** 5-year OS.

Ten studies reported 1-year OS, 13 studies reported 3-year OS and 6 studies reported 5-year OS. The heterogeneity was low for all categories (I^2^ = 0%), and the fixed effect model showed that 1-year OS (OR=0.92, 95%CI= 0.68~1.24, P=0.57) (**Figure 7A**), 3-year OS (OR=1.30, 95%CI= 1.00~1.68, P=0.05) (**Figure 7B**), 5-year OS (OR=1.38, 95%CI= 0.92~2.07, P=0.12) (**Figure 7C**), Which there no significant differences between the two groups.

### Subgroup analysis

N-PSM subgroup analysis showed differences in the results of lymph node metastasis and recurrence compared to that in non-subgroup analysis, whereas the other variables were consistent (**Table 3**). PSM subgroup analysis showed differences in the results of overall complications, while the other variables had similar results in both subgroup and non-subgroup analyses (**Table 3**). Therefore, the stability of our meta-analysis results is good (see **Supplement Figures S1–S21**). Results of previously published meta-analysis are summarized in supplement Table 4.

**Table 3:** The results of the meta-analysis are summarized of subgroup.

Subgroup	PSM					N-PSM				
Outcomes	Study	Cases	I^2^	MD/OR (95% CI)	P	Study	Cases	I^2^	MD/OR (95% CI)	P
Operating time	6	832	0.92	18.48 (-4.04, 41.01)	0.11	8	426	0.46	-0.86 (-28.22, 26.50)	0.95
Blood loss	5	588	0.65	-138.23 (-203.91, -72.54)	**<0.01**	7	368	0.72	-120.38 (-174.43, -66.33)	**<0.01**
Transfusion	5	902	0.11	0.47 (0.33, 0.67)	**<0.01**	4	252	0	0.30 (0.14, 0.62)	**0.001**
Lymph node dissection	6	766	0.74	0.49 (0.23, 1.06)	**0.01**	4	168	0.6	0.35 (0.10, 1.28)	0.11
Overall complications	3	480	0.45	0.83 (0.56, 1.25)	0.38	8	426	0	0.33 (0.21, 0.53)	**<0.01**
Severe complications	5	896	0.58	0.84 (0.57, 1.24)	0.38	6	342	0	0.60 (0.30, 1.19)	0.14
Biliary leakage	2	262	0.53	0.71 (0.24, 2.08)	0.53	4	256	0	0.72 (0.24, 2.12)	0.55
Hospital stay	6	833	0.99	-3.68 (-6.12, -1.24)	**0.003**	6	353	0.5	-1.77 (-2.87, -0.67)	**0.002**
R0	5	888	0.03	1.70 (1.12, 2.58)	**<0.01**	6	2618	0	1.47 (1.12, 1.93)	**0.006**
Number of lymph node	3	604	0.99	0.94 (-0.44, 2.32)	0.18	3	160	0.98	-5.52(-15.34, -4.30)	0.27
Resection margin	3	446	0.71	1.81 (-0.95, 4.56)	0.2	2	69	0	1.09 (-3.61, 5.78)	0.65
Lymph node metastasis	2	232	0	0.38 (0.17, 0.85)	**0.02**	5	228	0.04	0.76 (0.41, 1.40)	0.37
Recurrence	3	440	0	0.62 (0.43, 0.91)	**0.01**	6	250	0	0.79 (0.45, 1.38)	0.4
Intrahepatic recurrence	1	104	N	0.59 (0.34, 1.04)	0.07	4	153	0.26	0.88 (0.41, 1.90)	0.74
Extrahepatic recurrence	1	104	N	0.52 (0.24, 1.11)	0.09	4	153	0	0.91 (0.43, 1.92)	0.8
1 OS	6	953	0	0.78 (0.55, 1.11)	0.16	4	262	0.16	1.44 (0.80, 2.60)	0.23
3 OS	7	1001	0	1.25 (0.93, 1.68)	0.14	6	250	0	1.49 (0.85, 2.60)	0.16
5 OS	3	631	0.5	1.50 (0.93, 2.42)	0.1	3	138	0	1.12 (0.52, 2.40)	0.78
1 DFS	5	744	0.36	1.33 (0.95, 1.87)	0.1	3	197	0	0.86 (0.46, 1.58)	0.62
3 DFS	6	792	0.05	1.20 (0.87, 1.65)	0.27	5	185	0.44	1.02 (0.52, 2.02)	0.94
5 DFS	2	422	0.67	1.05 (0.23, 4.86)	0.95	2	73	0.71	0.56 (0.08, 3.92)	0.56

MD/OR, mean difference/odd ratio;N-PSM, Non-PSM.

Statistical significant results are shown in bold.

### Sensitivity analysis and publication bias evaluation

**Figure 8:**
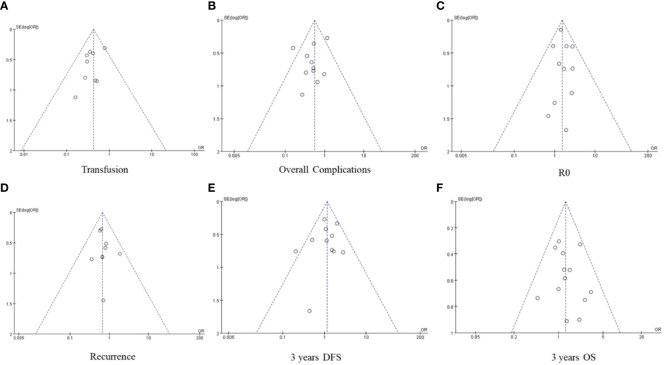
**(A)** transfusion **(B)** overall complication **(C)** R0 **(D)** recurrence **(E)** 3-year DFS **(F)** 3-year OS.

The results of operation time, blood loss, lymph node dissection, hospital stay, number of lymph node, retention margin, and 5-year DFS were highly heterogeneous. Exclusion of individual studies did not significantly affect the heterogeneity, and the results are consistent, indicating that our results are stable. After removing the study by Kinoshita et al. (35), the heterogeneity of 5-year DFS decreased significantly (I^2 =^ 35%), although further analysis with the fixed effect model did not indicate any significant difference between the Lap-ICC and open-ICC groups (OR=1.49, 95%CI=0.84~2.62, P=0.17). Funnel maps were plotted based on transfusion (**Figure 8A**), overall complications (**Figure 8B**), R0 (**Figure 8C**), recurrence (**Figure 8D**), 3-year OS (**Figure 8E**) and 3-year DFS (**Figure 8F**), which showed a symmetrical distribution of scatter points on both sides of the funnel. This suggested the lack of any obvious publication bias in this meta-analysis.

## Discussion

To the best of our knowledge, this is the latest meta-analysis and systematic review of laparoscopic and open hepatectomy outcomes in ICC patients with the largest sample size, the highest quality studies and the most comprehensive outcome indicators. Our findings indicate that Lap-ICC is superior to Open-ICC as far as some short-term outcomes are concerned, including less bleeding and shorter hospital stay. However, the rate of lymph node dissection was less in the Lap-ICC group. Furthermore, although laparoscopy was able to achieve a higher R0 resection rate and lower tumor recurrence, the two groups had similar DFS and OS rates at 1, 3 and 5 years.

The Liver Cancer Study Group of Japan recommends surgical resection for single tumors without regional lymph node metastasis (38). In addition, several guidelines also recommend palliative chemotherapy for bi- or multifocal ICC (39, 40). However, a recent cohort study reported similar long-term efficacy of surgical resection and chemotherapy for multifocal ICC, and a meta-analysis suggested that re-operation may partly improve the short-term and long-term outcomes of patients with recurrent ICC (41, 42). At present, open hepatectomy is the most common surgical method used for the treatment of ICC. It entails complete removal of the tumor while retaining sufficient liver volume, and requires regional lymph node dissection according to the latest guidelines of AJCC (43, 44). However, open hepatectomy is often traumatic and patients recover slowly. Although laparoscopy can reduce patient trauma and accelerate recovery to some extent, it is beset with challenges such as the difficulty in routine regional lymph node dissection, and increased risk of tumor implantation and metastasis in the pneumoperitoneum. Therefore, the choice of open versus laparoscopic hepatectomy is a controversial point in ICC.

Previous meta-analyses that compared open and laparoscopic hepatectomy for ICC (**Table 4**) differed in the conclusions compared to our study, which may be attributed to differences in tumor diameter, staging, lymph node dissection ratio, CA-199 level and other baseline characteristics. Therefore, we included 8 high-quality PSM studies for subgroup analysis to further ensure the reliability of our results. Laparoscopic hepatectomy was associated with less intraoperative bleeding and shorter hospital stay, which is consistent with the previous meta-analysis and also explains the lower blood transfusion rate in the Lap-ICC group. These observations are consistent with that of laparoscopic surgeries for colorectal and gastric tumors (45, 46). There was no difference in the operation time between the two groups. For centers that routinely perform laparoscopy, the duration of laparoscopy and laparotomy are similar (47, 48). Furthermore, lymph node dissection was rarely performed in the Lap-ICC group, which might have also reduced the operation time of laparoscopy comparable to that of open hepatectomy.

**Table 4:** summary of previous meta-analyses.

First author	Year	Number	Study types	Including studys	Registration	Outcomes	Results
Wei	2019	6	RS(6)	Uy 2015, Ratti 2015, Lee 2016,Wei 2017, Kinoshita 2019, Martin 2019	N	Major hepatectomy, Operating time, Blood loss, Pro portions of T1, Morbidity, R0 resection, Tumor size, Lymph node dissection, Lymph node metastasis, Recurrence,OS	Compared with Open-ICC, Lap-ICC has more R0 resections (RR = 1.08) but less major hepatectomies (RR = 0.69), less lymph node dissections (RR = 0.62) and smaller tumor size resected (WMD=-0.80cm). No significant difference was observed in other results.
Guerrini	2020	4	RS(4)	Uy 2015, Ratti 2015, Lee 2016,Wei 2017	N	Operation time, Major hepatectomy, Blood loss, Transfusion, Pringle maneuver, Hospital stay, complications, R0, Lymph node dissection, Recurrence	Compared with Open-ICC, Lap-ICC had lower blood loss (MD= −173.86 ml), less transfusion (OR =0.34), less need for Pringle maneuver (OR=0.17), shorter hospital stay (MD= − 3.77 day), and lessmorbidity (OR=0.44), lower rates of lymphadenectomy (OR=0.12), No significant difference was observed in other results.
Machairas	2020	8	RS(5), PSM(3)	Lee 2016,Wei 2017, Kinoshita 2019, Martin 2019. Zhu 2019, Ratti 2020, Haber 2020, Kang 2020	N	Major hepatectomy, Operating time, Blood loss, Transfusion, R0 resection, Number of retrieved lymph nodes, Overall complications, Major complications, Mortality, Hospital stay, 3 year-DFS, 3-year OS	Compared with Open-ICC, Lap-ICC had smaller tumors (MD=−1.17 cm), less major resections (RR=0.75), more R0 resections (RR=1.05), less lymph node dissections (RR=0.73), less blood loss (MD=−270.16 ml), less transfusion (RR=0.39), less overall morbidity (RR=0.58), shorter hospital stay (MD=−3.48 days). No significant difference was observed in other results.
Regmi	2020	8	RS(5), PSM(3)	Lee 2016,Wei 2017, Kinoshita 2019, Martin 2019. Zhu 2019, Ratti 2020, Haber 2020, Kang 2020	N	Tumor size, Operation time, Blood loss, Transfusion, Major hepatectomy, R0 resection, Number of resected LN, Number of positive LN, Hospital stay, Overall complications, Major complications, Mortality, 3 year-OS, 5 year-OS, 3year DFS, 5year DFS, Recurrence	Compared with Open-ICC, Lap-ICC had less blood transfusion (OR=0.32), higher R0 resection (OR), shorter length of stay (SMD=-0.40), less overall morbidities (OR=0.50), and less death due to tumor recurrence (OR=0.50), but Lap-ICC was associated with smaller ICC, fewer major hepatectomies, less LN dissection rate, and inferior 5-year OS (P < 0.05). No significant difference was observed in other results.
Ziogas	2021	8	RS(5), PSM(3)	Lee 2016,Wei 2017, Kinoshita 2019, Martin 2019. Zhu 2019, Ratti 2020, Haber 2020, Kang 2020	N	Overall complications, Severe complications, Operating time, Blood loss, Transfusion, Hospital stay, Lymph node dissection, Number of LN resected, R0 resection, Recurrence, OS, DFS	Compared with Open-ICC, Lap-ICC had lower overall complication (RR=0.64), less surgical lymphadenectomy (RR=0.74) and margin-positive resection (RR=0.78), higher recurrence-free rate (RR =1.24). No significant difference was observed in other results.
Zhao	2022	17	RS(9), PSM(8)	Uy 2015, Lee 2016,Wei 2017, Kinoshita 2019, Martin 2019. Zhu 2019, Ratti 2020, Haber 2020, Kang 2020, Wu 2020, Lee 2021, Hobeike 2021, Ma 2021, Chen 2021, Brustia 2022, Salehi 2022, Yang 2022	CRD42020164778	Operating time, Blood loss, Transfusion, LN dissection, Overall complication, Severe overall complication, Biliary leakage, Hospital stay, R0 resection, Number of lymph node, Resection margin, Lymph node metastasis, Recurrence, Intrahepatic Recurrence, Extrahepatic Recurrence, 1 year DFS, 3 year DFS, 5 year DFS, 1 year OS, 3 year OS, 5 year OS	Compared to Open-ICC, Lap-ICC had lower rates of lymph node dissection (OR=0.44) and metastasis (OR=0.58), along with less intraoperative bleeding (MD=-128.43 ml,) lower blood transfusion rate (OR=0.43), shorter hospital stay (MD=-2.75 day), higher R0 resection rate (OR=1.60), and lower tumor recurrence rate (OR=0.67). No significant difference was observed in other results.

Laparoscopic lymph node dissection may increase the risk of postoperative complications, tumor implantation and metastasis. Therefore, the lower rate of lymph node dissection in the Lap-ICC group may have reduced the incidence of lymph node metastasis compared to that in the open-ICC group. Zhang et al. recently showed that lymph nodes metastasis is an independent predictor of long-term survival of ICC. The AJCC Eighth Edition Cancer Staging also stipulated the number of lymph nodes dissection, which should not be less than 6 (13, 49). However, there is no difference in the number of lymph node dissection between the two groups. Although there are reports that laparoscopic lymphadenectomy increases the incidence of perioperative complications, several meta-analyses have shown that the overall incidence of perioperative complications is lower in the laparoscopic group compared to the open hepatectomy group (12, 14–18). We also detected lower incidence of overall perioperative complications in the Lap-ICC group compared to the open-ICC group, whereas the incidence of severe complications and bile leakage were similar in the two groups. However, PSM subgroup analysis showed that there was no difference in the frequency of overall complications, severe complications and bile leakage between the two groups. Therefore, it is possible that laparoscopy may not be beneficial to ICC patients in terms of perioperative complications.

R0 resection rate is a significant predictor of tumor recurrence and long-term survival of cancer patients. A previous meta-analysis reported higher R0 resection rate for laparoscopy compared to laparotomy, which can be attributed to the smaller tumors and better accessibility in the laparoscopy group (16–18). However, the number of cases in the study of Martin et al. (24) was significantly higher than that in the other studies included in the meta-analysis. Therefore, after removing the data of this study, there was no significant difference in the R0 resection rate between the two groups. Likewise, we further analyzed the R0 resection rate of the five latest PSM studies, excluded the influence of tumor diameter and other factors on the results, and found that the R0 resection rate was higher in the Lap-ICC group (OR=1.60, P=0.01), which was in line with the lower tumor recurrence rate observed in this group (OR=0.67, P=0.01). However, the intrahepatic and extrahepatic recurrence rates were similar in both groups. Although the tumor recurrence rate was lower in the Lap-ICC group, the 1-year, 3-year and 5-year DFS of the two groups were similar, which may be due to the longer follow-up time of laparotomy or the difference of follow-up time between the two groups. In addition, there was no significant difference in 1-year OS, 3-year OS and 5-year OS between the two groups. It shows that the higher R0 resection rate and lower recurrence rate after laparoscopic surgery can not be transformed into survival benefits.

Our study has some limitations that ought to be considered. First, the included studies were non-RCTs. In addition, the selection criteria of minimally invasive approach is highly restrictive (small tumors, generally less than 5 cm, far away from the hepatic hilus, no need for biliary reconstruction). However, the data of large hepatectomy with or without relevant biliary or vascular reconstruction *via* minimally invasive approach are still very few. Although we conducted PSM subgroup analysis to control the interference of these factors as much as possible, some factors (such as small sample size, tumor location, adjuvant radiotherapy and chemotherapy) may affect the results. Third, the results of the survival analysis may have been statistically deficient since we used OR for comparison. Nevertheless, our aim was to assess the long-term survival trends. Our findings will have to be validated with an RCT on large sample size.

To sum up, laparoscopic hepatectomy has better short-term outcomes in ICC patients compared to open hepatectomy, such as less bleeding, shorter hospital stay and higher R0 resection rate, while the long-term efficacy of both is similar.

## Author contributions

ZX, GFW and LZH conceived the study, had full access to all the data in the study, and take responsibility for the integrity of the data and the accuracy of the data analysis. ZX and GFW designed the search strategy and discussed with YJ, JKY and XQY, GJ, YMY, YQ and MTY performed study selection, data extraction and synthesis. ZX and GFW drafted and led on the writing of the manuscript. All the other authors participated in the analysis and interpretation of the data, revised the manuscript critically for important intellectual content and re-drafted some of its section. All the authors read and approved the final version of the manuscript, and agreed to be accountable for all aspects of the work to ensure its accuracy and integrity.
