# Supplementary figures and images for: Laparoscopic or open liver resection for intrahepatic cholangiocarcinoma: A meta-analysis and systematic review
Source: Front Oncol. 2023 Mar 1;13:1096714. doi: 10.3389/fonc.2023.1096714 (PMC10014898; doi:10.3389/fonc.2023.1096714)

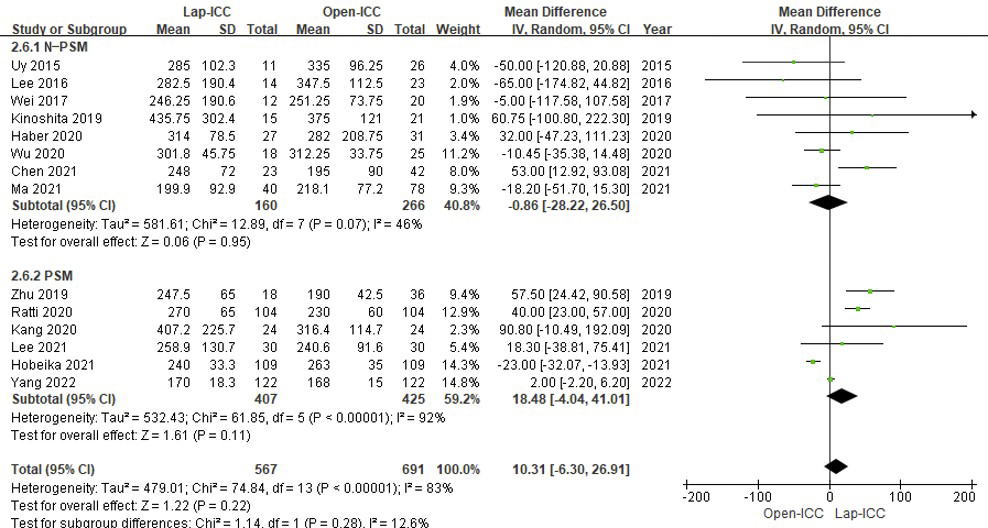

Supplement: Supplementary file 1 [file Presentation_1.zip › Supplement-new/new-Figure S1-operating time.jpg]

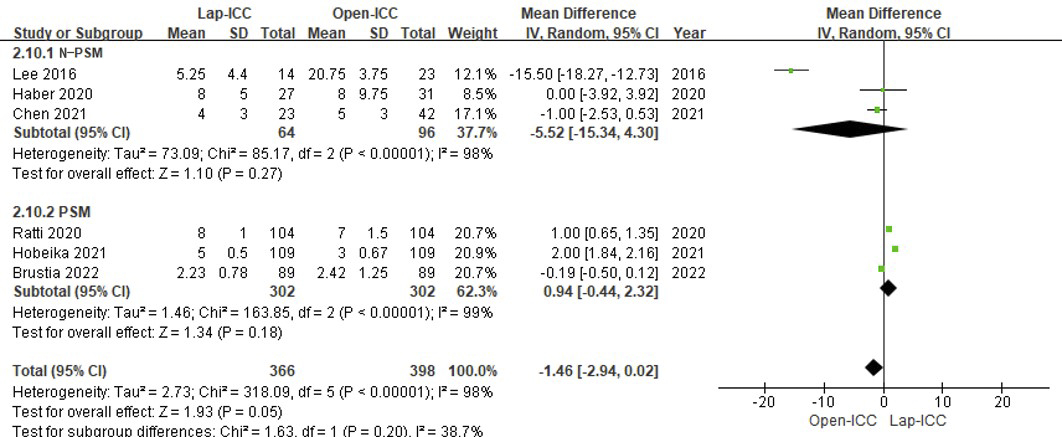

Supplement: Supplementary file 1 [file Presentation_1.zip › Supplement-new/new-Figure S10-number of retrieved lymph node.jpg]

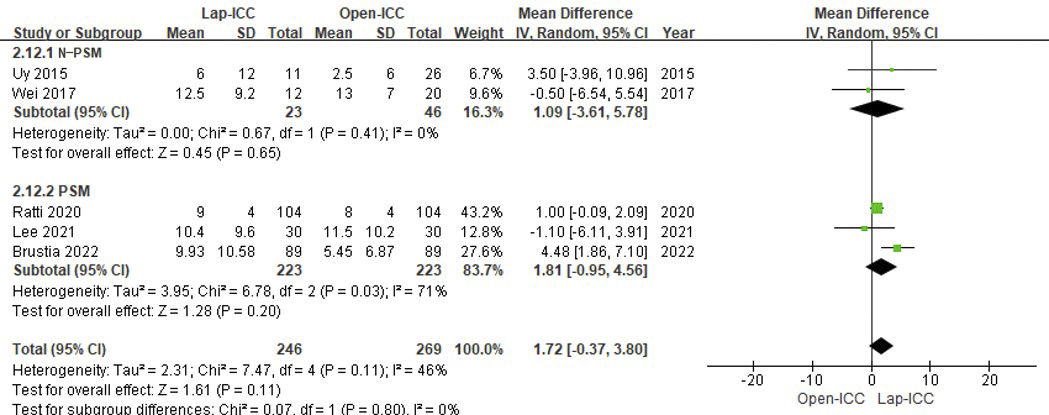

Supplement: Supplementary file 1 [file Presentation_1.zip › Supplement-new/new-Figure S11-resection margin.jpg]

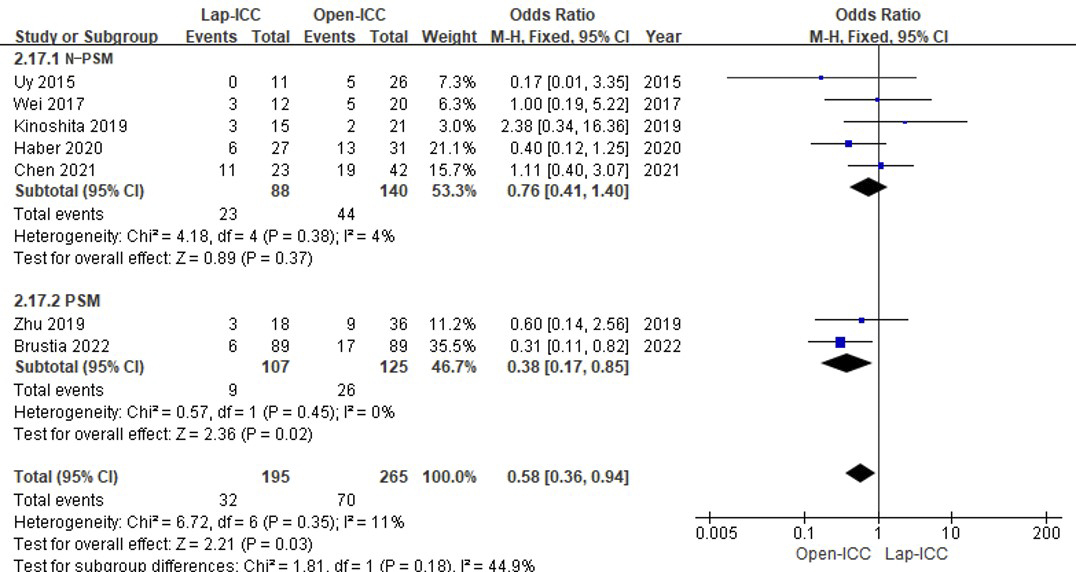

Supplement: Supplementary file 1 [file Presentation_1.zip › Supplement-new/new-Figure S12-lymph node metastasis.jpg]

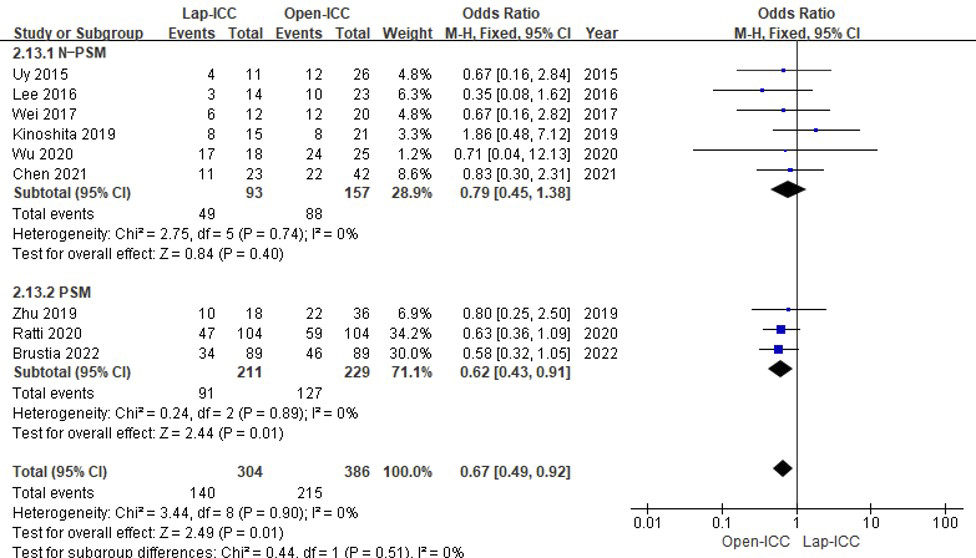

Supplement: Supplementary file 1 [file Presentation_1.zip › Supplement-new/new-Figure S13-recurrence.jpg]

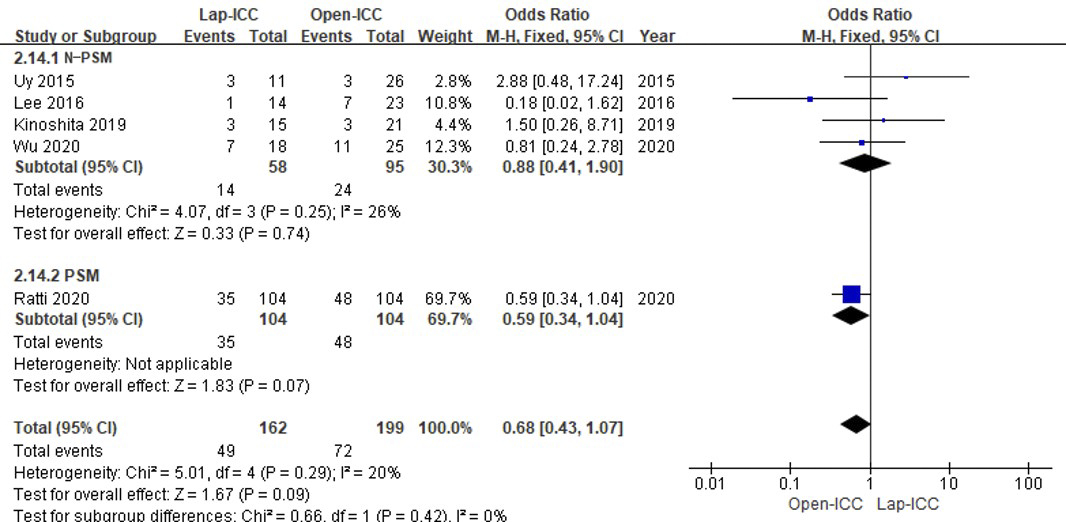

Supplement: Supplementary file 1 [file Presentation_1.zip › Supplement-new/new-Figure S14-intrahepatic recurrence.jpg]

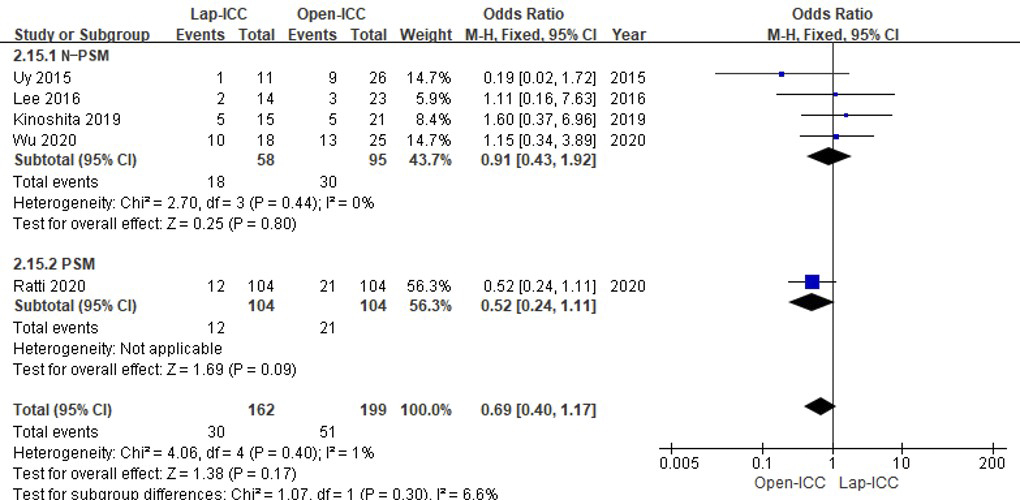

Supplement: Supplementary file 1 [file Presentation_1.zip › Supplement-new/new-Figure S15-extrahepatic recurrence.jpg]

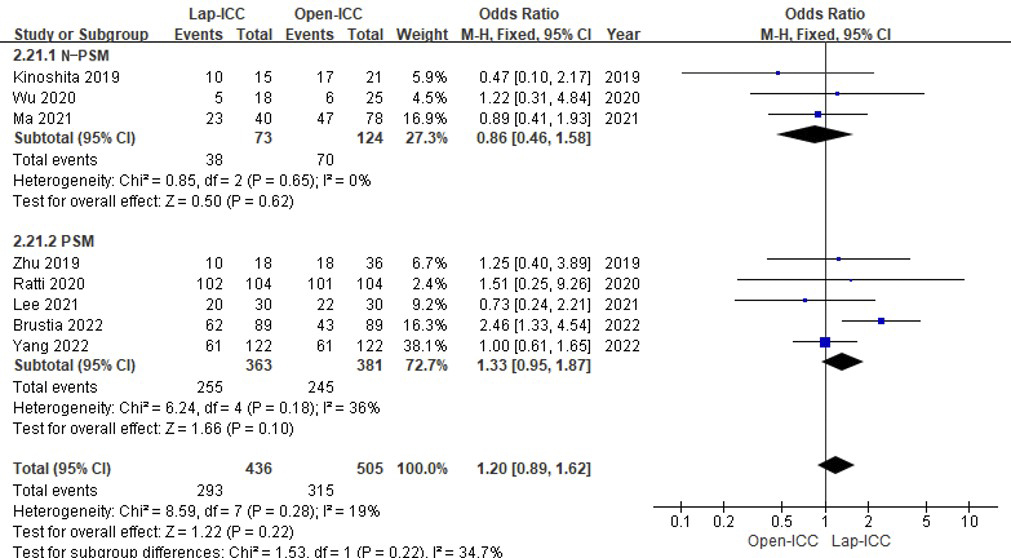

Supplement: Supplementary file 1 [file Presentation_1.zip › Supplement-new/new-Figure S16-1 year DFS.jpg]

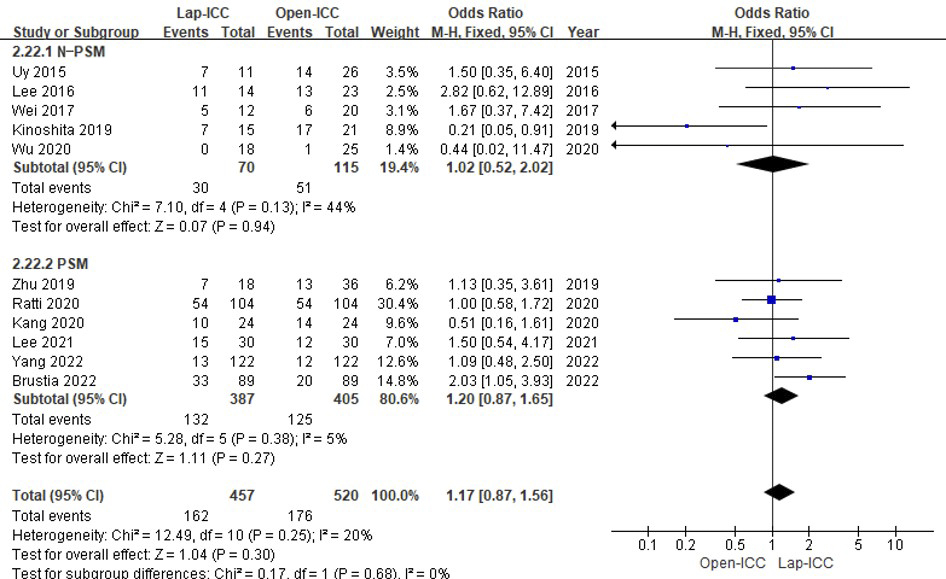

Supplement: Supplementary file 1 [file Presentation_1.zip › Supplement-new/new-Figure S17-3 year DFS.jpg]

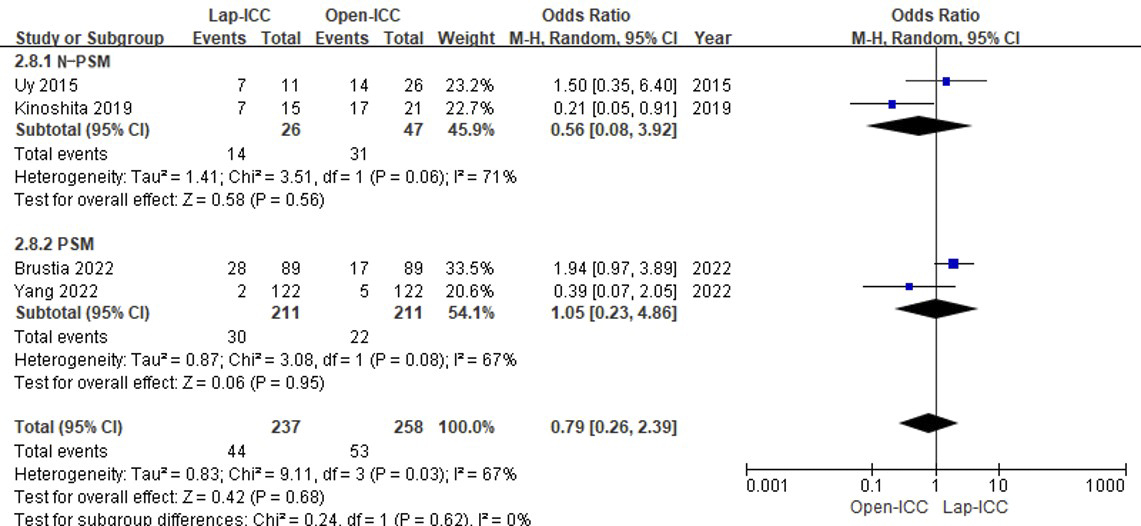

Supplement: Supplementary file 1 [file Presentation_1.zip › Supplement-new/new-Figure S18-5 year DFS.jpg]

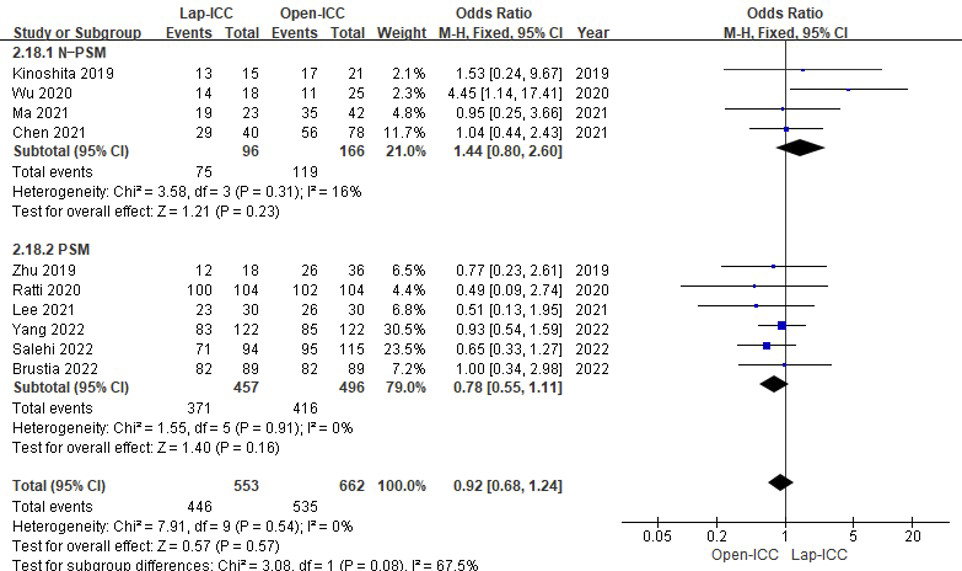

Supplement: Supplementary file 1 [file Presentation_1.zip › Supplement-new/new-Figure S19-1 year OS.jpg]

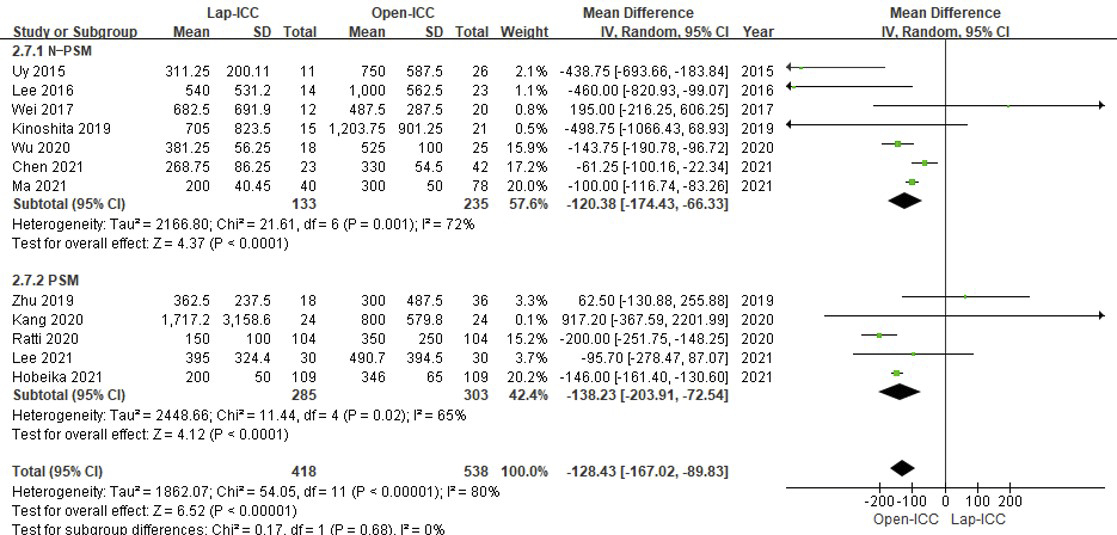

Supplement: Supplementary file 1 [file Presentation_1.zip › Supplement-new/new-Figure S2-blood loss.jpg]

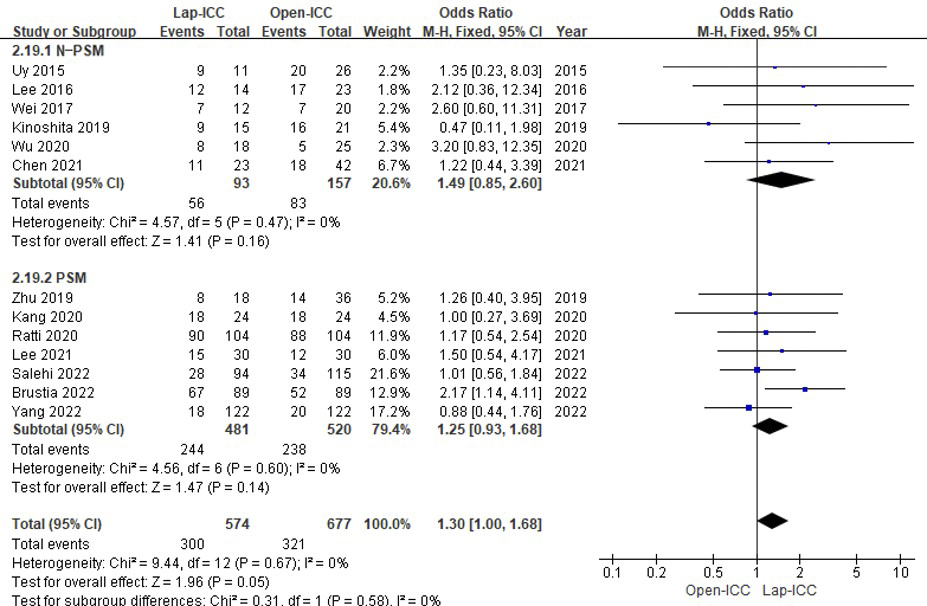

Supplement: Supplementary file 1 [file Presentation_1.zip › Supplement-new/new-Figure S20-3 year OS.jpg]

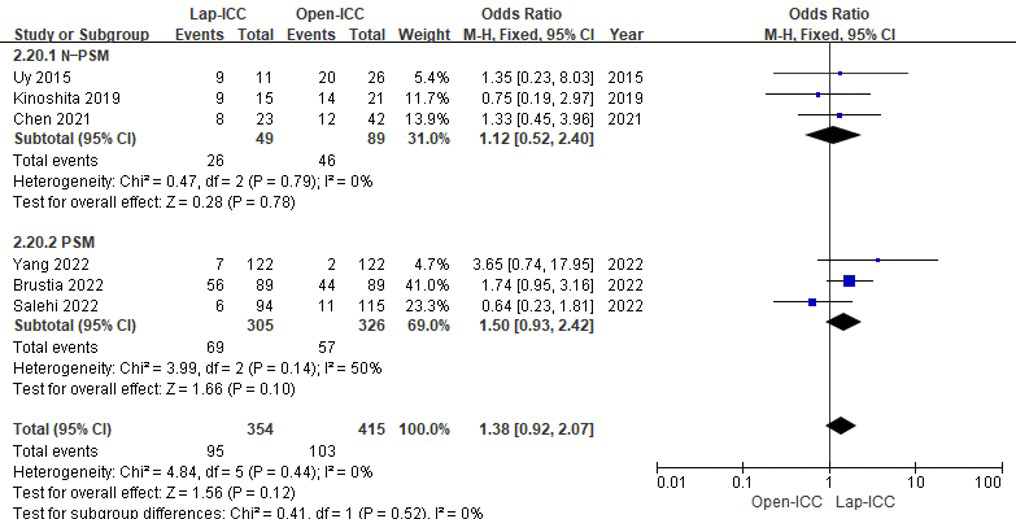

Supplement: Supplementary file 1 [file Presentation_1.zip › Supplement-new/new-Figure S21-5 year OS.jpg]

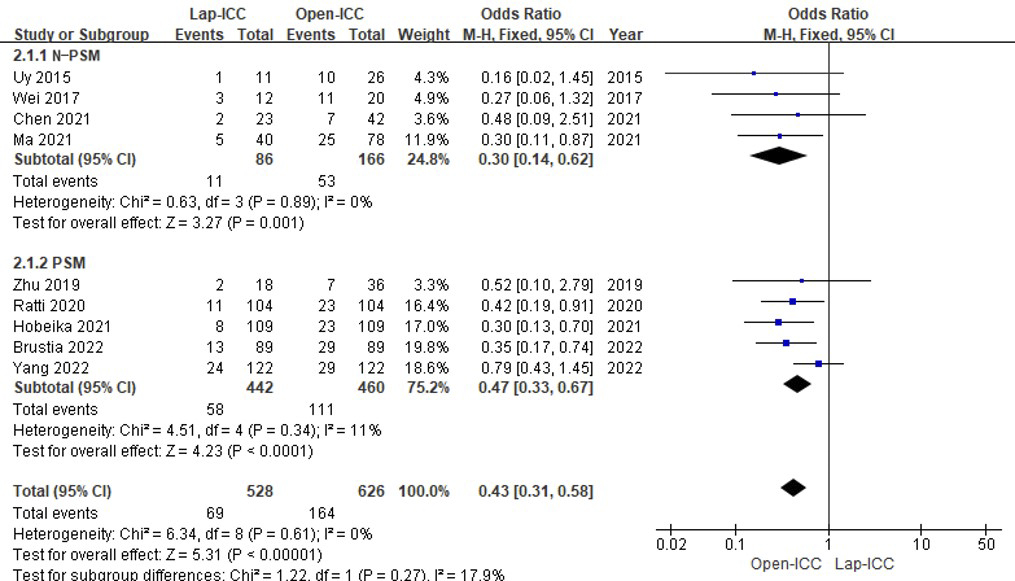

Supplement: Supplementary file 1 [file Presentation_1.zip › Supplement-new/new-Figure S3-transfusion.jpg]

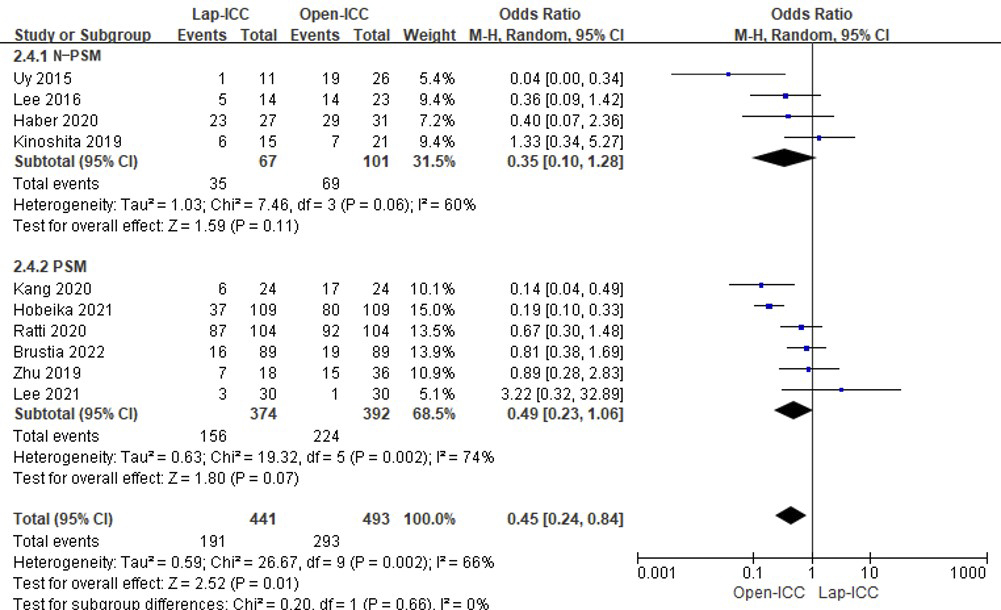

Supplement: Supplementary file 1 [file Presentation_1.zip › Supplement-new/new-Figure S4-lymph node dissection.jpg]

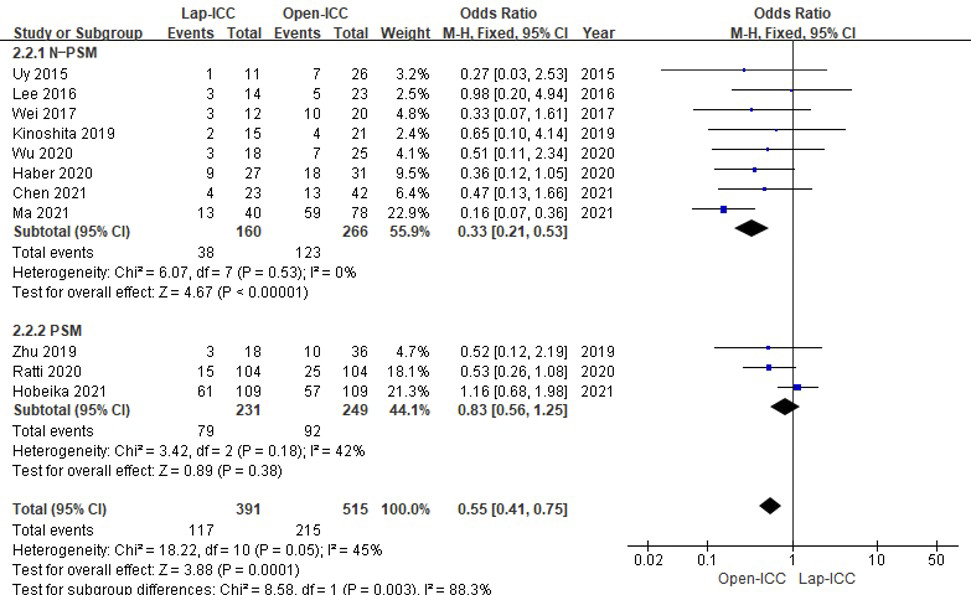

Supplement: Supplementary file 1 [file Presentation_1.zip › Supplement-new/new-Figure S5-overall complications.jpg]

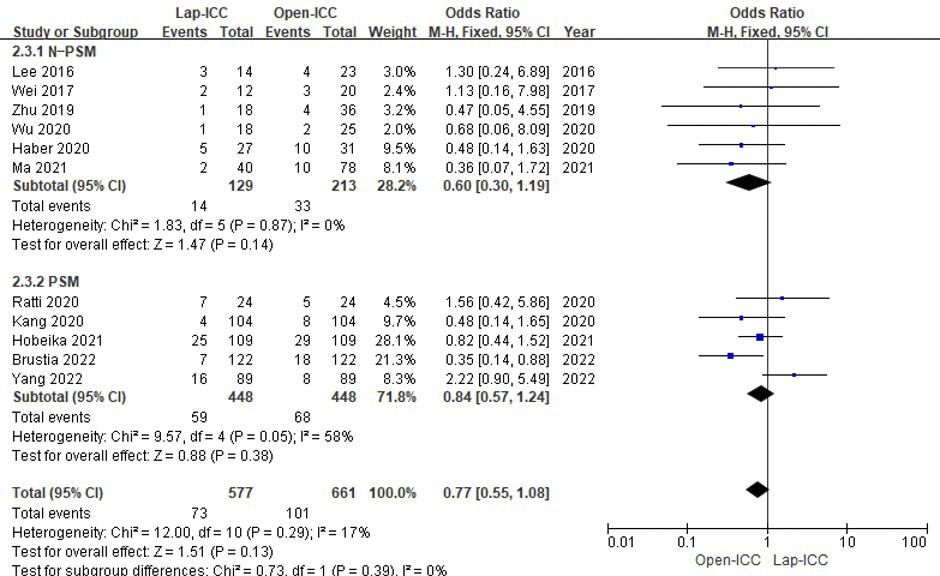

Supplement: Supplementary file 1 [file Presentation_1.zip › Supplement-new/new-Figure S6-severe complications.jpg]

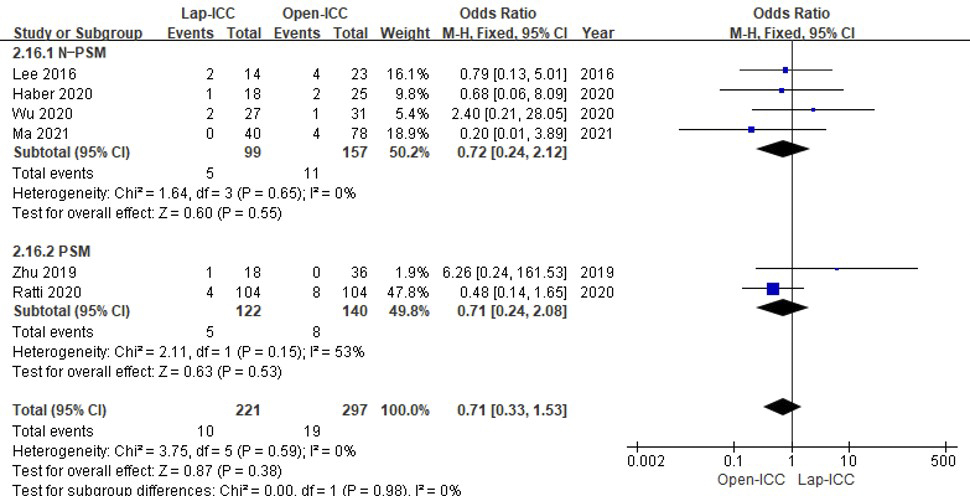

Supplement: Supplementary file 1 [file Presentation_1.zip › Supplement-new/new-Figure S7-biliary leakage.jpg]

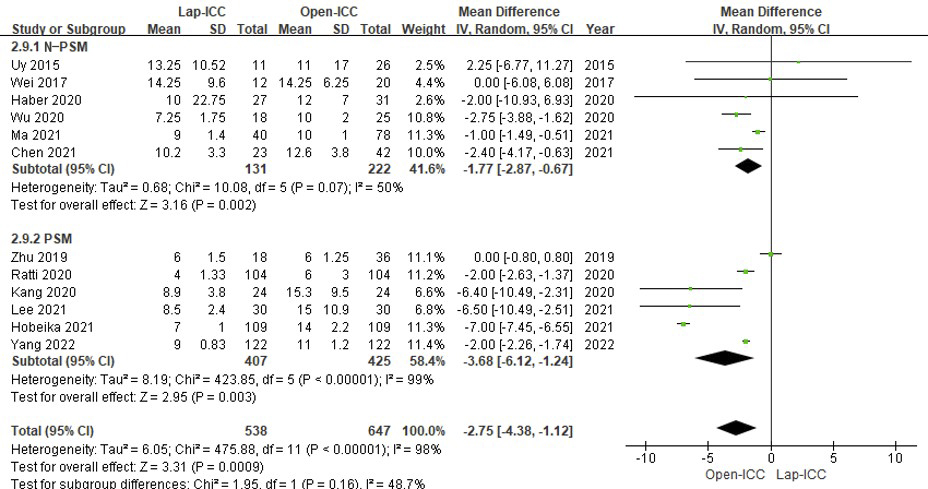

Supplement: Supplementary file 1 [file Presentation_1.zip › Supplement-new/new-Figure S8-hospital stay.jpg]

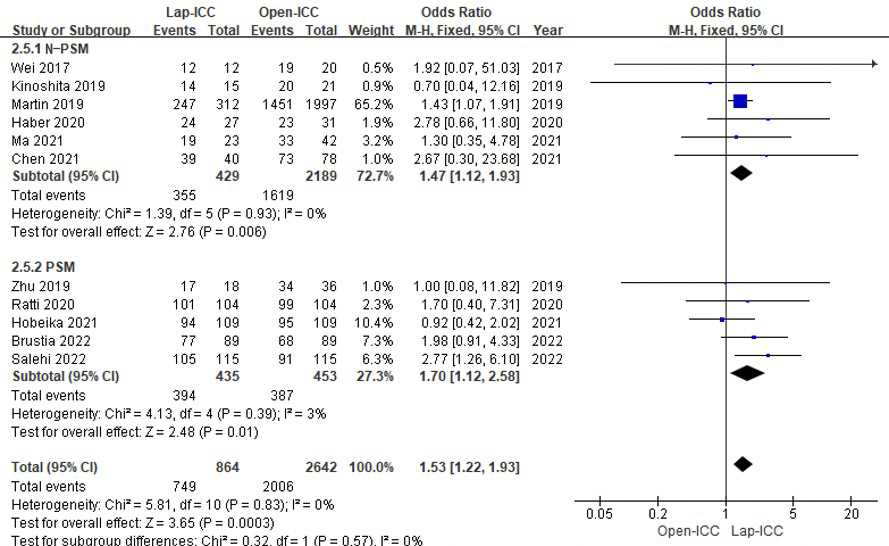

Supplement: Supplementary file 1 [file Presentation_1.zip › Supplement-new/new-Figure S9-R0.jpg]
